# Treatment with Mammalian Ste-20-like Kinase 1/2 (MST1/2) Inhibitor XMU-MP-1 Improves Glucose Tolerance in Streptozotocin-Induced Diabetes Mice

**DOI:** 10.3390/molecules25194381

**Published:** 2020-09-24

**Authors:** Zakiyatul Faizah, Bella Amanda, Faisal Yusuf Ashari, Efta Triastuti, Rebecca Oxtoby, Anny Setijo Rahaju, M. Aminudin Aziz, Maria Inge Lusida, Delvac Oceandy

**Affiliations:** 1 Department of Biomedical Sciences, Faculty of Medicine, Universitas Airlangga, Surabaya 60132, Indonesia; zakiyatul-f@fk.unair.ac.id (Z.F.); bella.amanda@fk.unair.ac.id (B.A.); faisalyusufashari@gmail.com (F.Y.A.); azizandro@gmail.com (M.A.A.); 2 Division of Cardiovascular Sciences, Faculty of Biology, Medicine and Health, University of Manchester, Manchester M13 9PT, UK; efta.triastuti@postgrad.manchester.ac.uk (E.T.); b.oxtoby@hotmail.com (R.O.); 3 Department of Pharmacy, Faculty of Medicine, Univesitas Brawijaya, Malang 65145, Indonesia; 4 Department of Anatomical Pathology, Faculty of Medicine, Universitas Airlangga, Surabaya 60132, Indonesia; anny_sr@fk.unair.ac.id; 5 Institute of Tropical Disease, Universitas Airlangga, Surabaya 60286, Indonesia; ingelusida@itd.unair.ac.id; 6 Department of Microbiology, Faculty of Medicine, Universitas Airlangga, Surabaya 60132, Indonesia

**Keywords:** diabetes mellitus, MST1/2, Hippo pathway, XMU-MP-1

## Abstract

Diabetes mellitus (DM) is one of the major causes of death in the world. There are two types of DM—type 1 DM and type 2 DM. Type 1 DM can only be treated by insulin injection whereas type 2 DM is commonly treated using anti-hyperglycemic agents. Despite its effectiveness in controlling blood glucose level, this therapeutic approach is not able to reduce the decline in the number of functional pancreatic β cells. MST1 is a strong pro-apoptotic kinase that is expressed in pancreatic β cells. It induces β cell death and impairs insulin secretion. Recently, a potent and specific inhibitor for MST1, called XMU-MP-1, was identified and characterized. We hypothesized that treatment with XMU-MP-1 would produce beneficial effects by improving the survival and function of the pancreatic β cells. We used INS-1 cells and STZ-induced diabetic mice as in vitro and in vivo models to test the effect of XMU-MP-1 treatment. We found that XMU-MP-1 inhibited MST1/2 activity in INS-1 cells. Moreover, treatment with XMU-MP-1 produced a beneficial effect in improving glucose tolerance in the STZ-induced diabetic mouse model. Histological analysis indicated that XMU-MP-1 increased the number of pancreatic β cells and enhanced Langerhans islet area in the severe diabetic mice. Overall, this study showed that MST1 could become a promising therapeutic target for diabetes mellitus.

## 1. Introduction

Diabetes mellitus (DM) is one of the major causes of death in the world [1]. A total of 463 million (9.3%) people worldwide were estimated to suffer from diabetes in 2019. This number is estimated to increase to 578 million (10.2%) in 2030 and 700 million (10.9%) in 2045 [2]. DM might induce multi-organ complications, such as retinopathy, neuropathy, cerebrovascular diseases, nephropathy, and cardiovascular diseases [3]. At present, the most commonly used treatment for DM is anti-hyperglycemic agents, that can reduce the detrimental effects of high blood glucose level [4]. However, this therapeutic strategy cannot stop the decline in the number of functional pancreatic β cells, which are important in the production and secretion of insulin. Discovery of new therapeutic strategies that can prevent the damage of pancreatic β cells due to DM will be useful in tackling this disease in the future.

There are two types of DM—type 1 DM (T1DM) and type 2 DM (T2DM). T1DM is caused by pancreatic β cell destruction mainly due to the autoimmune process. Its onset often occurs in children and young adults. Since the cause of T1DM is pancreatic β cell damage, the main treatment strategy for this condition is injection with insulin [5]. On the other hand, T2DM is caused by insulin resistance in the peripheral target organs as well as defective insulin secretion [6]. T2DM is normally associated with metabolic syndrome (obesity, hypertension, dyslipidemia). These conditions can change the molecular properties of the insulin receptor in the target tissues, such as adipose and skeletal muscle tissues. In contrast to T1DM, the main therapeutic strategy for T2DM is using anti-hyperglycemic agents that work by inducing insulin sensitivity (glitazones), reducing glucose production in liver (metformin) or enhancing insulin release by pancreatic β cells (sulfonylureas) [7].

Pancreatic β cell damage due to apoptosis plays an important role in both types of DM. In T1DM, β cell destruction occurs following an autoimmune reaction, which triggers activation of inflammatory cells and secretion of cytokines [8]. This will eventually activate the apoptotic pathways in the pancreatic β cells [9]. In T2DM, metabolic stress might lead to the activation of proinflammatory cytokines such as tumor necrosis factor-α (TNF-α) and interleukin-6 (IL-6) [10]. These cytokines might induce insulin resistance in the peripheral target organs, as well as contribute to the apoptotic cell death of pancreatic β cells [10].

One of the major signaling pathways that is heavily involved in the regulation of cell apoptosis, survival, and regeneration is the Hippo pathway [11]. The core components of the Hippo pathway in mammals include kinases—mammalian sterile 20-like kinase 1/2 (MST1/2) and large tumor suppressor 1/2 (LATS1/2), adaptor molecules (salvador (SAV1) and MOB kinase activator 1 (MOB1)), the transcriptional co-activators Yes-associated protein (YAP), and WW domain containing transcription regulator 1 (WWTR1/TAZ) [8,11]. Activation of the Hippo pathway by phosphorylation of the kinases results in YAP inactivation and hence reduces the expression of its target genes, which include anti-apoptotic and pro-survival genes. On the other hand, inhibition of the Hippo pathway result in YAP activation and eventually in induction of target genes expression [8,11].

Beside regulating the cell function via the canonical pathway, several members of the Hippo pathway might regulate apoptosis through alternative pathways. For example, MST1 was shown to be a powerful pro-apoptotic molecule through the modulation of B-cell lymphoma-extra-large (Bcl-xL) phosphorylation [12], regulation of the Forkhead box O1-NOXA (FoxO1-NOXA) pathway [13], and phosphorylation of histone H2AX [14]. MST1 is expressed in pancreatic β cells, making this kinase important in the pathophysiology of DM [15]. MST1 induces β cell death and impairs insulin secretion by initiating proteasomal degradation of pancreatic and duodenal homeobox 1 (PDX1), and β cell transcription factor [16]. In keeping with this finding, genetic inhibition of MST1 protected against β cell apoptosis and improved the diabetic phenotype in streptozotocin (STZ)-induced diabetes model in mice [17]. This finding prompted a notion that MST1 could be a novel therapeutic target for DM. Furthermore, a study using a cancer drug, neratinib, which was found to inhibit MST1 activity, indicated that pharmacological inhibition of MST1 was protective against the development of STZ-induced diabetes mellitus [17].

Recently, a novel molecule XMU-MP-1 was identified and characterized as a potent and specific inhibitor for MST1/2 [18]. It was demonstrated that treatment with XMU MP-1 in mice enhanced the proliferation of hepatocytes and small intestinal epithelial cells [18]. XMU MP-1 could also improve heart function and remodeling in a model of chronic cardiac pressure overload in mice, by decreasing apoptosis, fibrosis, and pathological hypertrophy [19]. Given the crucial role of MST1 in the pancreatic β cells and in the pathogenesis of DM, we hypothesized that treatment with XMU-MP-1 would produce beneficial effects by improving the survival and function of the pancreatic β cells. In this study, we tested this idea by performing in vitro and in vivo studies using the insulinoma cell line (INS-1) and an STZ-induced diabetes model in mice. We used the rat insulinoma cell line INS-1 832/13 in this study. We refer to this cell line as INS-1 cells, thereafter.

## 2. Results

### 2.1. The Effects of XMU-MP-1 Treatment on INS-1 Cells

**Figure 1:**
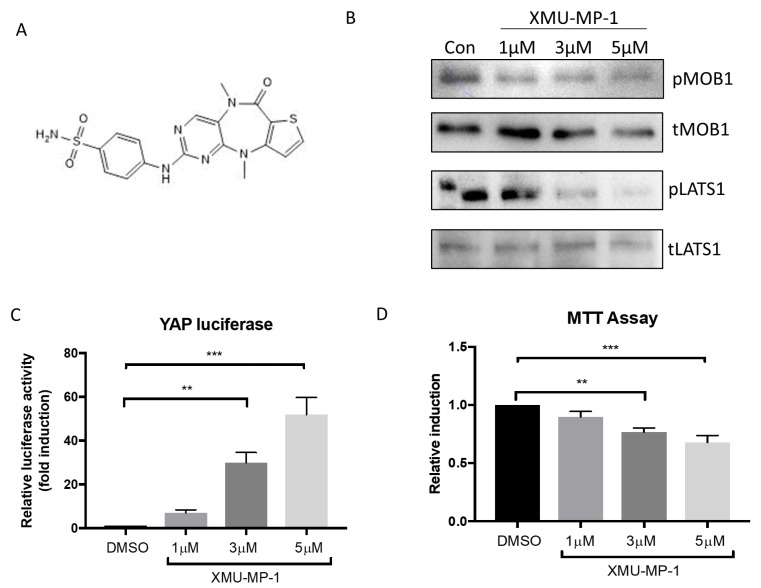
Effects of XMU-MP-1 treatment on INS-1 cells. (**A**) Molecular structure of XMU-MP-1. (**B**) XMU-MP-1 treatment reduced the phosphorylation of MOB1 and LATS1 in INS-1 cells. (**C**) Using an adenoviral-based YAP-luciferase reporter system, we detected a significant increase of YAP activity in INS-1 cells treated with XMU-MP-1, at doses of 3 μM and 5 μM for 24 h (*n* = 6 in each group). (**D**) MTT assay indicated that there was a reduction in cell survival following treatment with 3 μM and 5 μM of XMU-MP-1 for 24 h (*n* = 10 in each group). ** *p* < 0.01, *** *p* < 0.001.

To examine whether treatment with XMU-MP-1 (Figure 1A) modulates the Hippo signaling pathway in pancreatic β cells, we treated INS-1 cells with increasing doses of XMU-MP-1 (1–5 μM). As XMU-MP-1 targets the MST1/2 activity, we first looked at the phosphorylation of LATS1 and MOB1, which are known as downstream substrates of MST1/2. Western blot analysis showed reduction in LATS1 and MOB1 phosphorylation, as shown in Figure 1B. Further downstream, we explored if inhibition of MST1/2 in INS-1 cells would activate YAP, a major target effector of the Hippo Pathway. Using an established adenoviral-driven luciferase reporter system [19], we detected a significant and dose-dependent increase in YAP activity, following XMU-MP-1 treatment (Figure 1C). Together, these findings suggest that XMU-MP-1 treatment could inhibit MST1/2 activity in INS-1 cells, which subsequently enhanced the YAP activity.

In order to determine if the XMU-MP-1 treatment produced toxic effects in INS-1 cells, we performed an MTT assay to assess the level of INS-1 cell viability, following treatment with XMU-MP-1. Data shown in 1D indicated that at a lower dose (1 µM) of XMU-MP-1 did not induce significant cell death, however, cell viability was decreased after treatment with higher doses of XMU-MP-1 (3–5 µM). It is important to note that despite statistically significant reduction in INS-1 cell viability, there were still more than ~70% INS-1 cells that survived at 24 h, after treatment with 5 µM XMU-MP-1 (Figure 1C).

### 2.2. XMU-MP-1 Treatment Reduces STZ-Mediated Cell Death of INS-1 Cells

**Figure 2:**
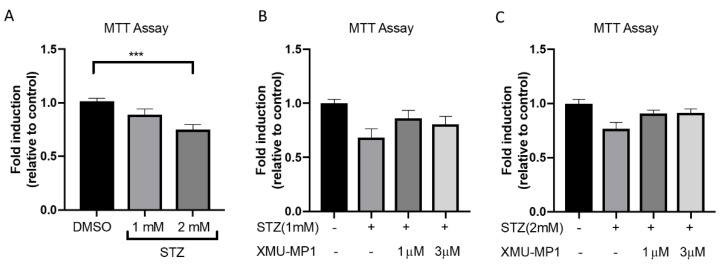
Effects of XMU-MP-1 treatment on INS-1 cell survival following streptozotocin treatment. (**A**) Treatment with STZ at 1–2 mM for 24 h resulted in a reduction in cell survival (*n* = 22–25 in each group, *** *p* < 0.001). (**B**) There was slight but non-significant increase in cell survival following XMU-MP-1 treatment in INS-1 cells, upon addition of 1 mM STZ (*n* = 18–32 in each group) or (**C**) 2 mM STZ (*n* = 11–21 in each group).

To investigate the effects of XMU-MP-1 treatment on STZ-induced toxicity in INS-1 cells, we assessed cell viability using an MTT assay. First, we performed experiments to determine the toxic dose of STZ in INS-1 cells. As expected, we found a dose dependent reduction in INS-1 cell viability following STZ treatment (1–2 mM for 24 h), with a statistical significance only observed, following treatment with 2 mM STZ (Figure 2A). We next analyzed the effects of XMU-MP-1 treatment, at doses of 1–3 µM, in addition to STZ administration. We observed more cell survival following treatment with 1 µM XMU-MP-1 on INS-1 treated with 1 mM STZ (Figure 2B) and in cells treated with 2 mM STZ (Figure 2C), although the differences did not reach statistical significance.

### 2.3. Effects of XMU-MP-1 Treatment in STZ-Induced Diabetic Mice

**Figure 3:**
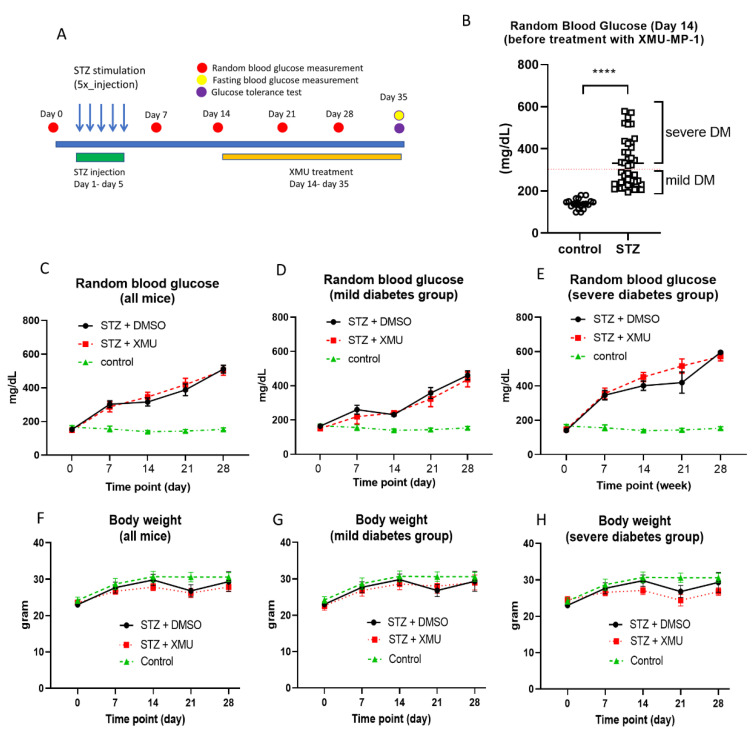
The effect of XMU-MP-1 treatment in STZ-induced diabetes model in mice. (**A**) Schematic diagram explaining the design of the experiments. (**B**) Analysis of random blood glucose (RBG) at day 14 after the first STZ injection (before XMU-MP-1 treatment); control, *n* = 20; STZ, *n* = 40). **** *p* < 0.0001. (**C**) RBG levels in all mice during the course of experiment (control, *n* = 10; STZ + DMSO, *n* = 20; STZ + XMU-MP-1, *n* = 20). (**D**) RBG levels in mice with mild diabetes (RBG 200–300 mg/dL at day 14) (control, *n* = 10; STZ + DMSO, *n* = 10; STZ + XMU-MP-1, *n* = 10). (**E**) RBG levels in mice with severe diabetes (RBG > 300 mg/dL at day 14) (control, *n* = 10; STZ + DMSO, *n* = 10; STZ + XMU-MP-1, *n* = 10). Body weight in (**F**) all mice, (**G**) mild diabetes mice, and (**H**) severe diabetes mice, during the course of the experiments.

Next, we conducted in vivo experiments using the STZ-induced diabetic mouse model, to examine the effects of XMU-MP-1 treatment. Figure 3A describes the design of the in vivo experiments. Wild-type Balb/c mice were injected with STZ at a dose of 50 mg/kg body weight (BW) per day, for 5 consecutive days. We analyzed the random blood glucose level before the STZ induction (day 0), and at day 7, 14, 21, and 28, following the first STZ injection. We treated diabetic mice with either XMU-MP-1 at 1 mg/kg BW/day or an equivalent volume of DMSO, for consecutive 21 days, starting from day 15 after the first STZ injection. At the end of the experiments (day 35), we measured the fasting blood glucose and performed glucose tolerance test (GTT).

We found that the blood glucose level was significantly elevated at two weeks, following the first STZ injection (Figure 3B). We classified these diabetic mice into two groups—mild diabetes group (random blood glucose 200–300 mg/dL) and severe diabetes group (random blood glucose >300 mg/dL). All diabetic mice in each group were randomly assigned to the experimental group (received XMU-MP-1 treatment) or the control diabetic group (injected with DMSO).

Random blood glucose was assessed every 7 days after the first STZ injection. We did not observe any difference in random blood glucose levels between the XMU-treated mice and the DMSO-treated mice, at any time points before and after the XMU/DMSO treatment (Figure 3C). Similarly, when we evaluated random blood glucose levels in the mild and severe diabetes groups, we did not find any difference between the XMU-treated and the DMSO-treated mice (Figure 3D,E).

Body weight was also monitored during the course of the experiments. Similar to the random blood glucose data, we did not observe any difference in body weight between the XMU-treated diabetic mice, DMSO-treated diabetic mice, and the normal controls (Figure 3F–H).

**Figure 4:**
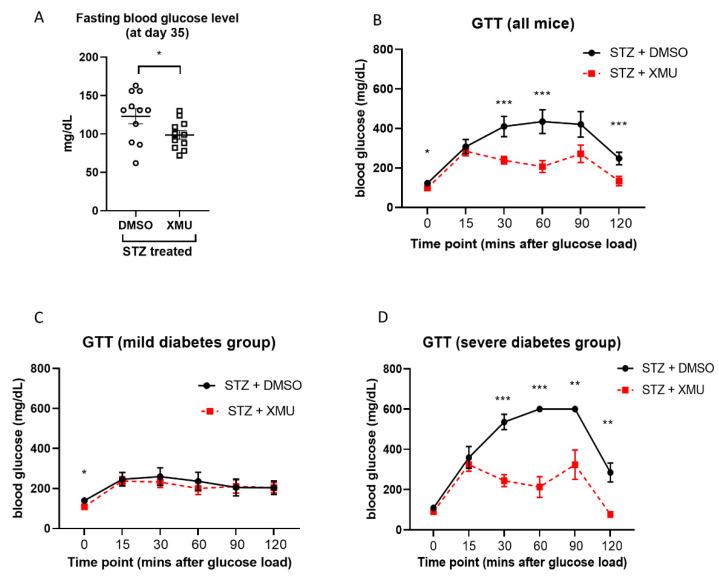
Analysis of blood glucose phenotype at the end of the experiments (day 35). (**A**) Analysis of fasting blood glucose (FBG) in all diabetic mice showed that mice treated with XMU-MP-1 exhibited lower FBG level (STZ + DMSO, *n* = 10; STZ + XMU-MP-1, *n* = 10). * *p* < 0.05. (**B**) Intraperitoneal glucose tolerance test (GTT) was performed by injecting mice with 1 g/kg BW glucose, followed by analysis of blood glucose at several time points as indicated in the graph. In all diabetic mice combined, we found that XMU-MP-1 treatment improved the blood glucose levels. (STZ + DMSO, *n* = 11; STZ + XMU, *n* = 11; * *p* < 0.05, *** *p* < 0.001). (**C**) Results of GTT in mild diabetes mice (RBG = 200–300 mg/dL at the start of treatment) (STZ + DMSO, *n* = 5; STZ + XMU, *n* = 5). (**D**) Results of GTT analysis in severe diabetes mice (RBG > 300 mg/dL at the start of treatment) (STZ + DMSO, *n* = 6; STZ + XMU, *n* = 6). * *p* < 0.05, ** *p* < 0.01, *** *p* < 0.001.

At the end of the experiments (day 35), we analyzed the fasting blood glucose and performed a glucose tolerance test. We found that the fasting blood glucose level was decreased in the XMU-treated group, compared to the DMSO-treated mice (Figure 4A). Furthermore, the glucose tolerance test (GTT) revealed that XMU-treated diabetic mice displayed a better response to bolus glucose injection (1g/kg body weight) compared to DMSO-treated diabetic mice (Figure 4B). In XMU-treated mice, the blood glucose started to decrease at 15 min after peritoneal glucose injection, whereas in the DMSO-treated diabetic group, the blood glucose stabilized only after 120 min after glucose injection. These data suggest that XMU-MP-1 might improve the response against glucose load in STZ-induced diabetic mice.

Next, we assessed the GTT results separately in the mild and severe diabetes groups. Evaluation of GTT results in these groups showed that the beneficial effects of XMU-MP-1 were observed only in the severe diabetic group (Figure 4C,D).

### 2.4. Protective Effect of XMU-MP-1 Treatment in Pancreatic Tissues

**Figure 5:**
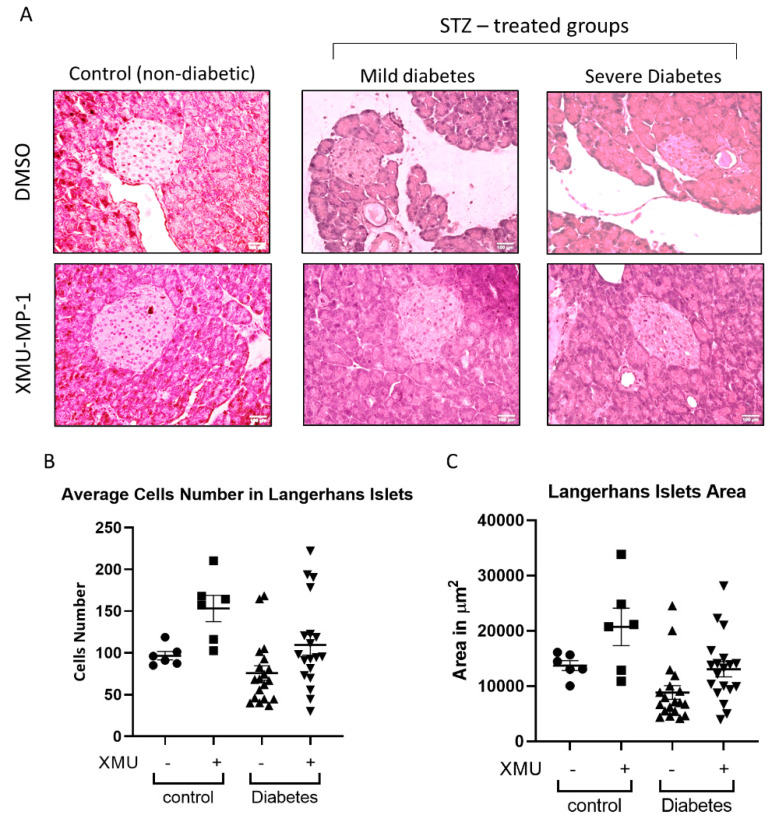
Histological analysis of pancreatic tissue sections. (**A**) Representative images of hematoxylin–eosin-stained pancreatic tissue sections showing Langerhans islets in all groups of experimental animals (scale bar = 100 µm). (**B**) The average number of cells in the Langerhans islets was examined in all groups. No significant difference in cell number was observed. (**C**) Analysis of the Langerhans islets surface area in all groups of mice. No significant difference in cell number was observed. (Control (non-diabetic), *n* = 6; control + XMU, *n* = 6; diabetic, *n* = 19, diabetic + XMU, *n* = 19).

To study the effects of XMU-MP-1 treatment on the histological structure of the pancreas, we examined hematoxylin-and-eosin-stained pancreatic tissue sections (Figure 5A). We focused our analysis on the structure of Langerhans islets by calculating the average cell number within one islet, and the average surface area of the Langerhans islets in the whole pancreatic sections. In the control mice (non-diabetic/no STZ), we found a trend of increased cell number and Langerhans islet area following XMU-MP-1 treatment, although the difference did not reach statistical significance (Figure 5B,C). We then analyzed the effects of XMU-MP-1 treatment in all diabetic mice (combined group of mild and severe diabetes). We did not find any significant effects of the XMU-MP-1 treatment in terms of the Langerhans islet surface area and the average cell number within the islet (Figure 5B,C).

**Figure 6:**
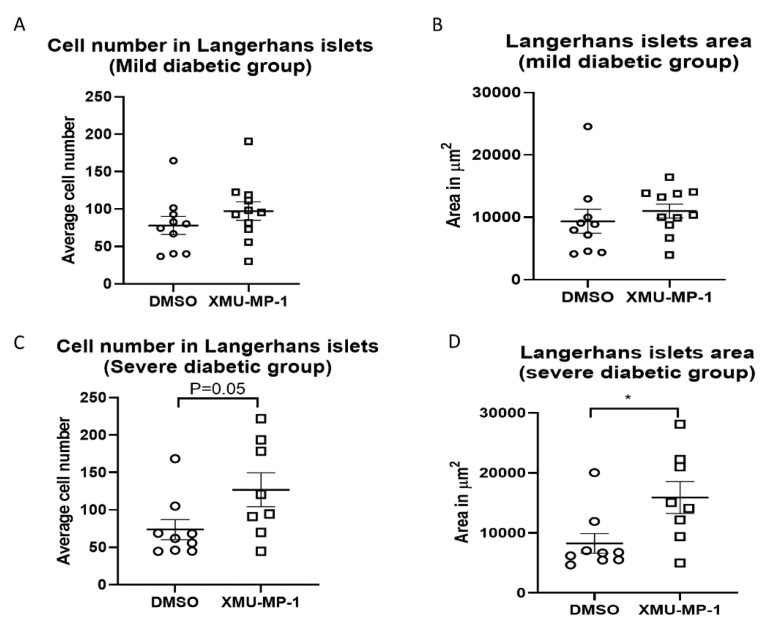
Analysis of Langerhans islets average cell number and surface area in specific diabetes groups. (**A**) Average cell number in the Langerhans islets and (**B**) the Langerhans islets surface area in mild diabetic mice. No significant difference was observed in this group of diabetic mice between the DMSO and XMU-MP-1 treatment (mild diabetes + DMSO, *n* = 10; mild diabetes + XMU MP-1, *n* = 11). (**C**) However, in the severe diabetic group there was a trend of a higher cell number and (**D**) a significant increase in the Langerhans islets surface area in the XMU-MP-1-treated mice, compared to the DMSO-treated diabetic mice (severe diabetes + DMSO, *n* = 9, severe diabetes + XMU MP-1, *n* = 8); * *p* < 0.05.

However, further analysis in each group of diabetic mice showed beneficial effects of XMU-MP-1 treatment, when given to the severe diabetic mice. Whilst there was no significant difference regarding the cell number and Langerhans islets surface area in the mild diabetic group (Figure 6A,B), we found a significantly enhanced cell number and islets area following the XMU-MP-1 treatment in the severe diabetic group (Figure 6C,D). These findings were in line with the GTT analysis and supported the idea that XMU-MP-1 was effective to improve the phenotype in the severe diabetic group but not in the mild diabetic mice.

## 3. Discussion

The main finding of this study was that treatment with the MST1/2 inhibitor XMU-MP-1 produced a beneficial effect in improving glucose tolerance in the STZ-induced diabetic mouse model. XMU-MP-1 treatment increased the number of pancreatic β cells and enhanced the Langerhans islet area in severe diabetic mice.

XMU-MP-1 is a potent and specific inhibitor of MST1 and MST2, which are key components of the Hippo pathway [11,20]. XMU-MP-1 is able to efficiently inhibit MST1/2 activities in various cell types including hepatocytes, the macrophage cell line, osteosarcoma cell line [18], rat primary cardiomyocytes [19], microglial cells [20], and breast cancer cell lines [21].

As for other kinase inhibitors, it is important to consider the selectivity of XMU-MP-1. A previous study reported XMU-MP-1 selectivity by performing a KINOMEscan analysis against a panel of 468 kinases [18]. The authors reported only very few kinases that displayed a high affinity to XMU-MP-1. It was reported that at the concentration of 1 µM, XMU-MP-1 inhibited two kinases to the same extent as the inhibition of MST1/2. These were aurora kinase A (AURKA) and phosphatidylinositol-4,5-bisphosphate 3-kinase catalytic subunit gamma (PIK3CG). AURKA is involved in regulating β cell proliferation [22], whereas PIK3CG was shown to positively modulate insulin secretion [23]. Based on these data, the expected phenotypes of AURKA and PIK3CG inhibition would be a reduction in pancreatic β cell function, which is contrary to our findings. Thus, we believe that the phenotypes of the diabetic mice following XMU-MP-1 in our model were more likely due to inhibition of MST1/2 kinases.

Here, we found that XMU-MP-1 was also effective in inhibiting MST1/2 and hence induced YAP activity in the pancreatic beta cell line INS-1. This finding is important because previous reports showed that genetic ablation of MST1 resulted in the protection against the development of STZ-induced diabetes mellitus in mice [16] via improvement of pancreatic β cell survival, and thus it is essential for studying the effects of XMU-MP-1 treatment in pancreatic β cells, as well as in the STZ-induced diabetes model.

Our in vitro data using the INS-1 cell line suggested that XMU-MP-1 treatment did not produce a significant effect in enhancing cell survival, following STZ treatment. However, we observed a slight trend of increasing cell survival against STZ-induced toxicity when XMU-MP-1 was added at a lower dose (1 μM). The absence of the protective effects of XMU-MP-1 in the in vitro model might be due to a possible toxic effect of XMU-MP-1 to INS-1 cells. When added at higher doses (3–5 μM), XMU-MP-1 caused a slight but statistically significant reduction of INS-1 cell survival. These effects might be cell-specific as toxicity was not reported when XMU-MP-1 was used in different cell types [18,19]. Although XMU-MP-1 is considered to be one of the most specific inhibitors of MST1/2, at higher doses, it also inhibits several other kinases such as AURKA, ABL1, DCAMKL1, and MAP3K2/3 [18]. Thus, the toxic effects of XMU-MP-1 on INS-1 cells might be due to an alteration of other signaling pathways and not solely due to the inhibition of the Hippo pathway.

In this study, we tested the effects of XMU-MP-1 treatment on an STZ-induced diabetes model in mice. STZ is widely used to induce experimental β-pancreatic cell damage, both in vitro and in vivo. STZ is a naturally occurring nitrosourea that is capable of inducing irreversible cell damage, through the production of free radicals and induction of DNA damage [24]. Its effects are specific to pancreatic β-cells, since it enters the cell via the glucose transporter 2 (GLUT2), which is abundantly expressed in the β- cells [25]. Since it targets the pancreatic β cells, the STZ-induced diabetes is often considered to be the most suitable model for T1DM. Although there were several limitations, such as possible toxic effects to other organs/cell types, STZ is still the most widely used model for T1DM [26].

In this study, we chose this model, because a previous report demonstrated that MST1 was involved in regulating pancreatic β cell apoptosis [16]. Active MST1 promotes pancreatic β cell apoptosis, a molecular process that resembles the progression of type-1 diabetes mellitus. Thus, inhibition of MST1 using a genetic or pharmacological approach should protect against the development of type 1 DM [16]. This was the main reason for the use of the STZ-induced diabetes model in this study. However, we believe that it is also important to study the effects of MST1 inhibition in the other type of DM, i.e., the type 2 DM. Several rodent models of T2DM are established including diet-induced T2DM (in which the animals were subjected to a high-fat and high-calorie diet for a period of time) and the genetically modified mouse models of T2DM [27].

A study using another inhibitor of MST1, Neratinib—which also showed MST1 inhibition to be protective—was also performed using STZ-induced diabetes mellitus [17]. Our data were consistent with these previous studies. We observed a significant improvement of glucose tolerance test (GTT) and fasting blood glucose in the XMU-MP-1-treated mice at the end of the experiments. However, analysis of random blood glucose (RBG) at various time-points did not show any changes between the XMU-MP-1 treated mice, compared to those treated with DMSO. The difference might be caused by the timing of the RBG test, which were carried out at random time-points. The GTT is considered to be a more sensitive assay to assess the physiological function of β cells in Langerhans islets, to respond to an acute increase of the blood glucose level. Analysis of the fasting blood glucose level supports the GTT data that XMU-MP-1 might produce beneficial effects in the STZ diabetes model. Interestingly, our data indicated that the positive effect of XMU-MP-1 treatment was observed in the severe diabetes group of mice, suggesting that the protective and possibly regenerative effects of XMU-MP-1 was more prominent if there was a significant damage of the pancreatic cells. Taken together, our data might suggest an improvement of pancreatic β cell function after XMU-MP-1 treatment, although it was only limited to the severe diabetic mice, when they were challenged to a high glucose load during GTT.

Our finding, at least in part, was in line with previous studies that used gene deletion and MST1 inhibitor neratinib to inhibit MST1 function [16,17]. However, the beneficial effects of XMU-MP-1 in preventing the extent of STZ-induced diabetes did not seem to be as strong as those of neratinib or in the genetic knockout models. This was probably due to some degree of toxicity of XMU-MP-1 in pancreatic β cells.

So, how does XMU-MP-1 improve pancreatic β cell function in the severe STZ-induced diabetic mice? Histological analysis revealed an increased number of cells within the Langerhans islets that eventually expanded the Langerhans islet area in mice treated with XMU-MP-1. There are several possibilities to explain this phenotype. XMU-MP-1 induces cell proliferation; it reduces cell death by inhibition of apoptosis; or a combination of both. In other cell types, e.g., hepatocytes and intestinal cells, it is understood that both genetic ablation of MST1/2 and pharmacological inhibition using XMU-MP-1, resulted in increased cell proliferation [18,28,29]. In neonatal cardiomyocytes, XMU-MP-1 also induced cell proliferation, although this effect was not observed in adult cardiomyocytes, which are known to be terminally differentiated and mitotically inactive cells [19]. In contrast, tissue-specific knock out of MST1 and MST2 in pancreatic tissue resulted in the opposite phenotype, i.e., reduction of pancreatic size, which was associated with morphological defects [30,31], suggesting a critical role of the Hippo/YAP pathway in the pancreas during embryonic development. These findings showed that the role of the Hippo pathway might be organ- and context-specific and the effects of the MST1/2 pharmacological inhibition in the pancreas might not simply be due to induction of cell proliferation. Indeed treatment with MST1 inhibitor, neratinib, resulted in β cell mass recovery and attenuation of cell apoptosis, but it did not affect cell proliferation [17]. In contrast, following STZ stimulation, MST1 KO mice showed both a reduction of apoptosis and an increase in cell proliferation that eventually enhanced the β cell mass [16]. Thus, it is possible that XMU-MP-1, besides promoting β cell survival (as indicated in our in vitro model), also induced proliferation of the pancreatic β cells in vivo.

One of the most important factors in drug development is to understand the safety, toxicity, and possible side effects. It is known that inhibition of the core components of the Hippo pathway, which results in induction of the YAP activity, is associated with increased cell proliferation and survival. Previous observations using MST1/2 inhibitor (XMU-MP-1) and YAP/TAZ activator (TT-10) suggested increases in hepatocytes, cardiomyocytes, and small intestinal epithelial cell proliferation [18,32]. These reports might suggest possible carcinogenic effects of these compounds, when used for a longer term. However, our own observation on using XMU-MP-1 to treat cardiac hypertrophy in mice showed that there were no adverse effects in the heart, kidney, or liver [19]. Histological analysis as well as evaluation of serum markers of liver and kidney function indicated no significant damage in these major organs, following XMU-MP-1 treatment [19].

Overall, our study adds to the growing body of evidence that targeting pancreatic β cell survival and regeneration could become a new strategy for the treatment of diabetes mellitus. There are several molecular targets and compounds that were studied in this field [17,33,34]. Our data support the idea that the key components of the Hippo signaling pathway, in this case MST1/2, could become a target for diabetes therapy. Further studies are required to define the exact mechanism(s) as well as evaluate the toxicity and the off-target effects of the inhibitor. Equally important, studies using different models of diabetes, e.g., the high-fat-diet-induced diabetes model, also need to be conducted to understand if MST1/2 inhibition is also effective in controlling this type of pathological condition.

## 4. Materials and Methods

### 4.1. INS-1 Cell Culture

The INS-1 cells were obtained from Sigma and are widely used as a model to study pancreatic beta-cell functions in vitro [35]. INS-1 cells were cultured and maintained in a T-175 culture flask with a growth medium containing 500 mL of RPMI 1640 with no L-Glutamine (Gibco #11534446), 10% fetal bovine serum (Gibco #11550356), 100 U/mL of penicillin/streptomycin (Gibco #11528876), 50 µM of 2-mercaptoethanol (Gibco #11528926), 1 mM of sodium pyruvate (Gibco #12539059), 2 mM of L-Glutamine (Gibco #15410314), and 10 mM of HEPES (Gibco #12509079). The medium was changed every two days or when the cells reached 80–85% confluency. After that, the INS-1 cells were passaged to several T-175 flasks and maintained in the 5% CO_2_ incubator at 37 °C. For experimental purposes, we seeded the INS-1 cells on 24-well plates (10^5^ cells per well for YAP luciferase and MTT assays) and 6-well plates (5 × 10^5^ cells per well for protein extraction).

### 4.2. Yes-Associated Protein (YAP) Luciferase Assay

The measurement of the transcriptional activity of YAP was conducted by utilizing the GAL4-TEAD-UAS luciferase reporter system, as described previously [19]. The INS-1 cells were seeded at a density of 10^5^ cells/well on 24-well plates and were kept in the 5% CO_2_ incubator at 37 °C for 24 h. On the following day, the cells were transfected with both adenoviruses expressing GAL4-TEAD and UAS-luciferase. The treatments (DMSO as control, 1 µM, 3 µM, and 5 µM of XMU-MP-1) were administered concomitantly with the adenoviruses. After 24 h of incubation, the cells were washed with PBS (Phosphate Buffer Saline) and lysed with 1× cell culture lysis buffer (Promega #E1531), at room temperature for 20 min. A total 20 µL of the cell lysate was mixed with 50 µL of luciferase substrate (Promega #E151A) in a luminometer tube. The luciferase signal was detected using the LB9507 luminometer.

### 4.3. Western Blot

Protein expression levels were examined by Western blot. RIPA buffer was used to extract total protein from the INS-1 cells. Cells were seeded on 6-well plates, at a density of 5 × 10^5^ cells/well, then treated with DMSO, 1 µM, 3 µM, or 5 µM of XMU-MP-1 for 24 h. The protein concentration was quantified using the Pierce BCA assay kit system (Thermo Scientific #23225). A total of 20 µg of protein samples with 6x Laemmli buffer were loaded into SDS-polyacrylamide separating gel (8% for LATS1 detection and 12% for MOB1 detection). An electrophoresis system was used to separate the proteins based on the molecular size. After transferring the proteins onto nitrocellulose membrane and blocking the membrane with 5% skim milk, the membrane was incubated with primary antibodies—anti-phospho-MOB1 (Cell Signaling Technology, #8699S), anti-total-MOB1 (Cell Signaling Technology, #13730S), anti-phospho-LATS1 (Cell Signaling Technology, #9157S),and anti-total-LATS1 (Protein Tech #17049-1-AP), overnight at 4 °C. After washing in TBST buffer, the membrane was incubated with the secondary antibody (anti-rabbit IgG, HRP-linked antibody from Cell Signaling Technology #7074S), for an hour, at room temperature. The ECL Western blotting detection reagent (Amersham Biosciences #RPN2232) was used to visualize the protein bands in a ChemiDoc XRS+Imaging system. The band intensities were quantified using the Image Lab software (3.0, Bio-Rad Laboratories, Inc., Hercules, CA, USA).

### 4.4. INS-1 Cell Survival Experiments

The INS-1 cells were treated with 1–2 mM of STZ (Sigma Aldrich #18883-66-4) for 24 h, to induce cell damage. DMSO-treated cells were used as a negative control. In the treatment groups, XMU-MP-1 (1–3 µM) was added concomitantly with STZ. Cells were then incubated at 37 °C for 24 h. MTT solution (3-(4,5-dimethylthiazol-2-yl)-2,5-diphenyltetrazolium bromide, Sigma) at a concentration of 0.5 mg/mL was used to detect the normal cell metabolites, by producing purple crystals at the bottom of the plates. The cells were incubated with the MTT solution for two hours at 37 °C. When the crystals appeared, they were dissolved using a solubilization solution containing isopropanol and 0.1 M HCl (100 µL/well). The absorbance of the dissolved crystals in the solubilization solution was measured using a Multiscan Ascent microplate reader (Thermo Labsystems Inc., Philadelphia, PA, USA), at 500 nm wavelength.

### 4.5. In Vivo Experiments

Animal experiments were performed in compliance with the principles for the ethical treatment of animals in research, as described in the Government Regulation of Republic of Indonesia No. 95/2012 and in accordance with the guidelines for the care and use of animals in biomedical research (National Guidelines on Health Research Ethics 2011, Ministry of Health, Indonesia). The experimental procedures were approved by the Research Ethics Committee, Faculty of Medicine, Universitas Airlangga.

We used wild type male Balb/c mice, which were obtained from UD Wistar, Yogyakarta, a certified laboratory animal breeder supplier. We used 12-weeks-old mice with an average body weight (BW) of 24.22 ± 1.53 g, at the start of the experiments. Mice were housed in the experimental animal facility within the Institute of Tropical Disease (Universitas Airlangga) in standard housing conditions for laboratory animals. Mice were maintained on a 12-h light/dark cycle in a controlled temperature of 19–22 °C and humidity of 40–65% for 1 week, before the experiments were started. Mice were fed with standard chow diet BR501.

To generate a model of diabetes mellitus, mice were injected with STZ at a dose of 50 mg/kg BW/day, for 5 consecutive days. We analyzed random blood glucose (RBG) at basal levels before STZ injection (day 0), and at day 7, 14, 21, and 28, following the first STZ injection. The criterion used for diabetes was RBG level more than 200 mg/dL, at day 14 after the first STZ injection. At day 14, the mice were divided into 5 groups: (i) control group; (ii) diabetic mice with RGB less than 300 mg/dL and treated with XMU-MP-1; (iii) diabetic mice with RGB less than 300 mg/dL and treated with vehicle (DMSO); (iv) diabetic mice with RGB more than 300 mg/dL and treated with XMU-MP-1; and (v) diabetic mice with RGB more than 300 mg/dL and treated with vehicle (DMSO). XMU-MP-1 at a dose of 1 mg/kg BW/day or equal volume of vehicle (DMSO) were administered intraperitoneally, starting at day 15 after the first STZ injection, for a total of 21 days. At the end of the experiments (day 35), mice were sacrificed following intraperitoneal glucose tolerance test (GTT) and fasting blood glucose measurement.

### 4.6. Random Blood Glucose Measurement

Random blood glucose measurement was performed on blood samples obtained from the mouse tail vein. Glucose level was measured using a glucometer kit (One Touch Select; Lifescan; Malvern, PA, USA), following the protocol recommended by the manufacturer.

### 4.7. Intraperitoneal Glucose Tolerance Test

For the intraperitoneal GTT, mice were fasted for 12 h overnight. The following morning, mice were injected intraperitoneally with 40% glucose solution (Otsuka, Otsu, Japan), at a dose of 1 g glucose/kg body weight. Blood glucose levels were measured using a glucometer test, before glucose injection, and at 15, 30, 60, 90, and 120 min after glucose injection.

### 4.8. Histological Analysis of Pancreatic Tissues

Pancreas tissues collected at the end of the experiment were fixed using 4% normal buffered formalin for 24 h. Tissues were processed overnight using a Krisme automated tissue processor and were then embedded in paraffin wax. The histological sections were prepared at 5 µM thickness, using a rotary microtome (Leica 2125, Chicago, IL, USA). Analysis of the Langerhans islets surface area and cell number were conducted using an Olympus BX-41 microscope with 400× magnification. Images were analyzed using the ImageJ software (v1.52, NIH, Bethesda, MD, USA).

### 4.9. Data Analysis

Data are presented as mean ± standard error of the mean (SEM). Statistical analysis was performed using the GraphPad Prism software (v8.4.3, GraphPad Software, San Diego, CA, USA). One-way analysis of variance (ANOVA) was used to compare significance among groups. If ANOVA produced a significant value of F (*p* < 0.05) and there was no significant variance in homogeneity then the post-hoc multi comparison analysis (Tukey′s) were conducted. For comparison between two groups, Student′s *t*-test was used. Multiple *t*-test analysis was used to compare IPGTT data at each specific time-point; *p* value < 0.05 was considered to be significant.
